# Effects of Timed Frontal Plane Pelvic Moments During Overground Walking With a Mobile TPAD System

**DOI:** 10.1109/TNSRE.2022.3213207

**Published:** 2023-01-30

**Authors:** Danielle M. Stramel, Antonio Prado, Serge H. Roy, Heakyung Kim, Sunil K. Agrawal

**Affiliations:** Department of Mechanical Engineering, Columbia University, New York, NY 10027 USA; Department of Mechanical Engineering, Columbia University, New York, NY 10027 USA; Delsys/Altec Inc., Natick, MA 01760 USA; Department of Physical Medicine and Rehabilitation, The University of Texas Southwestern Medical Center, Dallas, TX 75390 USA; Department of Mechanical Engineering, Columbia University, New York, NY 10027 USA, Department of Rehabilitation and Regenerative Medicine, Columbia University Medical Center, New York, NY 10032 USA

**Keywords:** Index Terms—, Biomechanics, force control, human in the loop, rehabilitation robotics, robot control

## Abstract

Robotic gait training may improve overground ambulation for individuals with poor control over pelvic motion. However, there is a need for an overground gait training robotic device that allows full control of pelvic movement and synchronizes applied forces to the user’s gait. This work evaluates an overground robotic gait trainer that applies synchronized forces on the user’s pelvis, the mobile Tethered Pelvic Assist Device. To illustrate one possible control scheme, we apply assistive frontal plane pelvic moments synchronized with the user’s continuous gait in real-time. Ten healthy adults walked with the robotic device, with and without frontal plane moments. The frontal plane moments corresponded to 10% of the user’s body weight with a moment arm of half their pelvic width. The frontal plane moments significantly increased the range of frontal plane pelvic angles from 2.6° to 9.9° and the sagittal and transverse planes from 4.6° to 10.1° and 3.0° to 8.3°, respectively. The frontal plane moments also significantly increased the activation of the left gluteus medius muscle, which assists in regulating pelvic obliquity. The right gluteus medius muscle activation did not significantly differ when frontal plane moments were applied. This work highlights the ability of the mobile Tethered Pelvic Assist Device to apply a continuous pelvic moment that is synchronized with the user’s gait cycle. This capability could change how overgroundrobotic gait training strategiesare designed and applied. The potential for gait training interventions that target gait deficits or muscle weakness can now be explored with the mobile Tethered Pelvic Assist Device.

## I. Introduction

THE pelvis plays an essential role in overground ambulation as it is the intermediate segment between the torso and the lower limbs. Not only do the muscles proximal to the pelvis assist in regulating the center of mass during gait, but the hip musculature, including the gluteus medius muscles, provide frontal plane stability for the pelvis during walking and single stance [1], [2], [3], [4], [5]. Therefore, the coordination and strength of the hip musculature are critical for maintaining balance during overground ambulation.

An individual’s ability to ambulate independently directly impacts their quality of life [6]. Improving an individual’s coordination and strength at the pelvic level could positively impact their gait for those with atypical or irregular gait patterns. For stroke survivors, lateral pelvic tilt while standing is highly correlated with weight-bearing asymmetry [7]. For children with diplegic cerebral palsy (CP), Kirkwood et al. showed that individuals with gross motor function classification system (GMFCS) level II had significantly reduced pelvic obliquity than level I, highlighting the need for frontal plane rehabilitation strategies for those individuals [8]. For individuals with hip osteoarthritis (OA), in-phase and anti-phase coordination rates between the lumbar and pelvic segments are altered [9], and decreased range of motion and pelvic obliquity asymmetry are present [10]. While these works show that multiple patient populations exhibit reduced pelvic obliquity, more work is needed to study whether correcting this improves functional movement.

Strengthening the hip musculature and improving pelvis-trunk-lower limb coordination could benefit many individuals. However, the requirement for multiple physical therapists to work with various segments of the human body simultaneously can be challenging [11], so robotic devices have been developed to assist physical therapists. Introducing robotic devices for gait training allows us to explore the scientific application of forces, trajectories, and prescribed variability to various motions to magnify the potential of gait training. Robotic gait training devices are designed with specific gait training goals in mind and thus vary significantly in architecture design [12] and control implementation [13].

### A. Prior Works

Each robotic gait training device’s architecture allows control of one or more specific joints, and not all devices are designed to allow or control pelvic motion. The pelvis has six degrees of freedom (DOF), three translational and three rotational, and many devices have been used to control and study pelvic motion. Some devices, like the treadmill-based MUCDA and overground RAPBT, focus only on controlling the translational movement of the pelvis [14], [15]. Other devices, like the footplate-based Healbot T and the overground BAR, control only the DOFs in the horizontal plane, i.e., the anteroposterior and mediolateral translation of the pelvis and the horizontal rotation [16], [17]. Mun et al.’s overground robotic walker for pelvic motion support [18] controls pelvic translations and horizontal rotation while passively allowing pelvic tilt and obliquity. The treadmill-based PAM achieves all DOF except the anteroposterior tilt, and so do both overground NatureGaits and AssistOn-Gait systems [19], [20], [21]. The treadmill-based TPAD achieves complete control over the six DOFs [22]. Many of these devices utilize an architecture comprised of two articulated end effectors located at each lateral pelvic extreme, like the treadmill-based ALTACRO [23] and the overground PA [24]. While various combinations of DOFs can allow the study of different complex pelvic motions, some robotic devices solely focus on activating the frontal pelvic obliquity, like the treadmill-based RGR Trainer [25] and the soft exoskeletal HAA [26]. Once a robotic device’s active and passive DOFs have been established, a complementary control must be implemented to assist or train gait movements.

Whether a device is assistive or rehabilitative depends on how it interacts with an individual. For example, in the exoskeletal HAA, a torque is applied to assist the hip abductor, as this torque is applied in parallel to the wearer’s hip abductor muscles [26]. However, a force or moment can be applied in opposition to the corresponding musculature to resist and potentially rehabilitate a weaker muscle. This was the methodology of our TPAD work by Kang et al., which applied a downward force on the pelvis, leading to an increase in soleus muscle peaks after training [27]. Some robotic platforms can be both, depending on the workspace of applicable forces and moments and the direction or timing of the applied pelvic forces. To fully tailor these assistive or rehabilitative forces, information about the user’s gait is required.

To target and train coordination of the pelvis, robotic devices can be articulated to control specific pelvic motions. Knowing where the individual is within their gait cycle is also imperative, as the prescribed motions or forces should change with gait progression. The way the gait cycle is used within the control of a robotic device can be discrete [28], [29], [30] or continuous [25], [31]. The gait cycle is broken down into sections for many discrete applications, like stance, swing, pre-swing, etc., and different loadings or motions are prescribed. Finite state machines (FSM) use inertial measurement units (IMU) [29] or load cell [28] data to determine the present gait phase. For a more continuous force or movement profile, a continuous estimation of the gait cycle percentage can be output from a normalized state space and a reference trajectory [25] or a machine learning algorithm in real-time [31]. These real-time gait cycle segmentation methods, coupled with the articulation of control of pelvic motion, advance the ability to train specific movements and muscles.

### B. Motivation and Novelty

Though the aforementioned robotic devices include fine control of pelvic motion, the rigid structures add inertia to the users, altering natural overground gait. Cable-driven parallel devices, like the TPAD [22], can manipulate the pelvis without adding mass to the individual or constraining natural movement. However, complete control over all pelvic DOFs with minimal restriction during overground gait has yet to be achieved. There is a need for this type of robotic architecture, but more work must be done to control the pelvis using real-time information from the user. Customized force magnitudes and directions are crucial to investigating training paradigms targeting specific gait deficits and muscle weaknesses.

Our group’s TPAD can apply three-dimensional forces and moments to the pelvic center. However, the external motion capture system, high-power electronics, and large frame restrict device portability, so a treadmill is used to facilitate ambulation. Multiple design challenges needed to be considered to achieve complete portability and accommodate overground walking using the TPAD technology. An external motion capture system cannot localize the pelvis within the workspace, which is necessary to calculate cable tensions. All electronics must be battery operated, so the high-power motors cannot be used. The overall size and weight of the device must be small enough to fit through a door and be maneuverable by the user. On top of these, the device structure and cable layout must be sturdy enough that when cable tensions are applied, the device does not shift as a result, but that the pelvis is manipulated with respect to the device. By tackling the design adjustments required to make the TPAD portable, the ability of the TPAD to apply targeted three-dimensional forces can be translated to overground ambulation.

This work evaluates an overground cable-driven gait training device that can apply variable forces and moments to the pelvis that are synchronized with the user’s gait in real-time, the first of its kind to do so. This parallel platform is built upon a posterior rollator and utilizes a waist belt worn around the user’s pelvis as its end effector. The direction, magnitude, and duration of the forces and moments applied to the waist can be controlled. The flexibility of applied pelvic forces allows the mTPAD device to be either assistive or rehabilitative depending on the selected force and moment scheme. In this work, an assistive moment is evaluated, as the moment applied is in the opposite direction of the moment created by gravity force acting at the center of mass about the stance leg hip joint. This moment strategy is evaluated as it may be beneficial for individuals with hip drop in the future - but this must first be evaluated for controller efficacy. The controller for this device uses the predicted gait cycle percentage of the user in real-time to determine the pelvis’s applied force and moment profile. The gait cycle percentage is predicted by the DeepSole system, a pair of instrumented insoles and a machine learning algorithm developed by our lab. By combining the mTPAD platform and the DeepSole system [31], we can apply a frontal plane moment to the individual’s pelvis in real-time while ambulating overground. Many force profiles could be implemented on one or more DOFs using the mTPAD, but we will first look at altering frontal plane pelvic obliquity in this work. In this way, we can highlight one of the many possible control schemes; a control scheme that could be used in the future to target a specific gait need that affects many individuals with weak hip musculature.

## II System Design

### A. System Setup

**Fig. 1.**
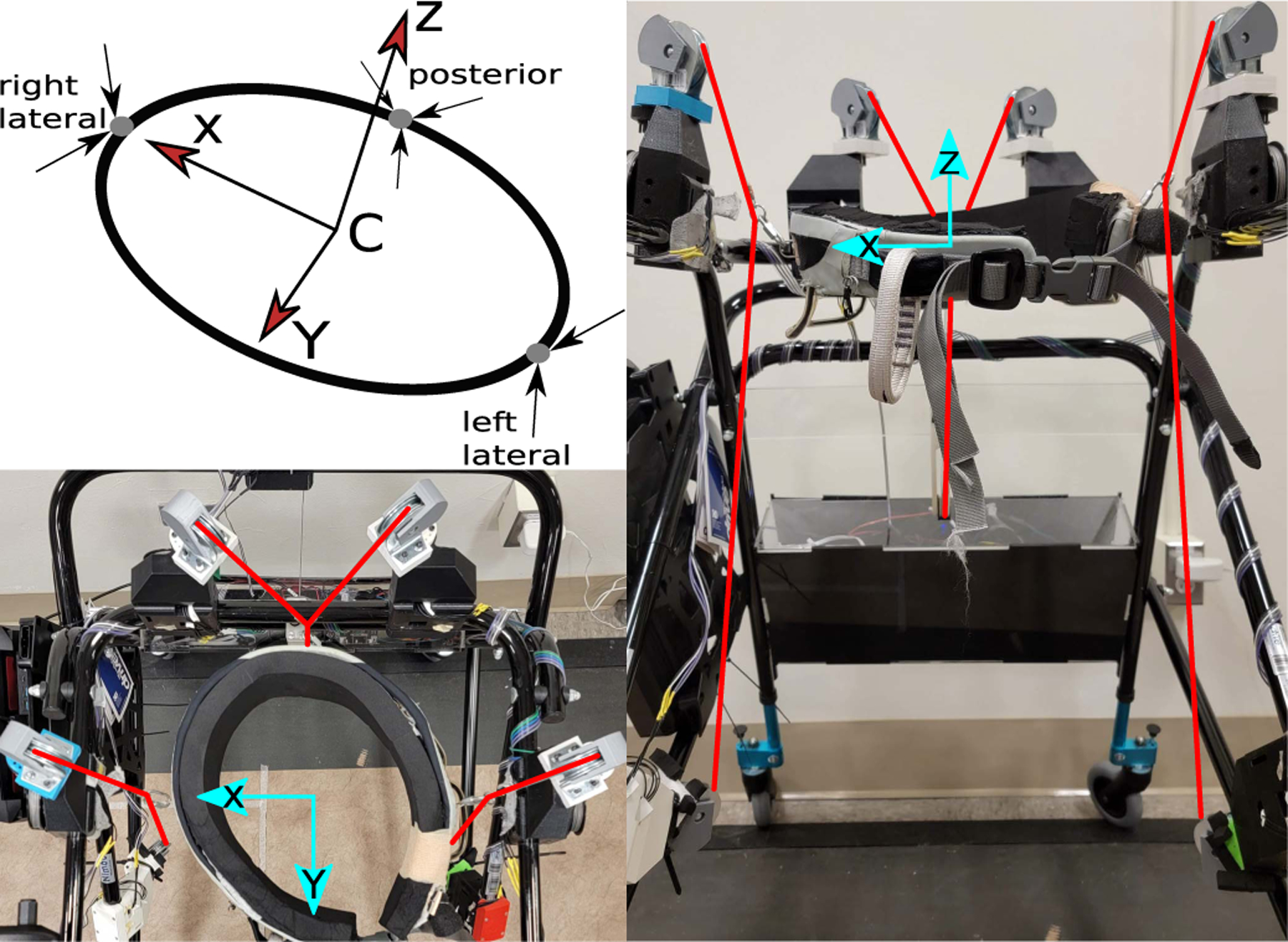
mTPAD architecture and representative pelvic coordinate frame. The upper left image illustrates the local pelvic coordinate frame used when applying pelvic forces. The left and right lateral points have two cables, while the posterior point has three. The local x-axis is aligned with the user’s frontal plane, and the z-axis is aligned with their vertical axis. More details on this can be found in [32]. The lower left shows a topdown view of the mTPAD with a pelvic belt. Red lines highlight the seven cables used. The x and y axes are shown in blue, with the z-axis coming out of the page. The right image shows a front view of the walker. The same illustrations highlight the seven cables and x and z axes, with the y-axis coming out of the page.

The timed pelvic moments in this experiment were applied using the mTPAD [32], a parallel, cable-driven system with seven actuated cables, as shown in Fig. 1. The frame of this device is an off-the-shelf posterior rollator (NIMBO, Inspired by Drive, California). This selection ensures that the device’s size and weight remain manageable for users to propel the device forward as they ambulate. Rather than the high-power motors used by the TPAD, Dynamixel servo motors (XM430W350-R, ROBOTIS, Seoul, South Korea) are used to provide the tension in each cable. These servo motors, coupled with a 3D-printed cable spool, can apply nearly 70 N per cable. Although the TPAD incorporates external tension sensors in line with each cable to have closed-loop control over cable tensions, these external sensors are not used in the mTPAD, as they would be difficult to route in such a tight pelvic workspace. Therefore, the mTPAD utilizes an open-loop control of the servo torques.

The most considerable challenge of mobilizing the TPAD was the localization of the pelvis. The TPAD relies on a VICON motion capture system to track markers located at specific points on the individual’s pelvic anatomy to track the pelvic center in real-time. However, these systems are expensive and limit the usable workspace of the robotic device. Therefore, a novel way of localizing the pelvic center with respect to the mTPAD frame is necessary. A forward kinematics approach that uses the cable lengths is implemented to solve this problem. Although this method is less accurate, the errors in the *x*, *y*, and *z* directions were on average −0.17 cm, 0.79 cm, and −0.54 cm, respectively [32].

Even with each of these alterations to the TPAD to create the mTPAD, the mTPAD can still apply specific forces to the user’s pelvis [32]. This force is applied using seven cables that route from the mTPAD to the pelvic belt worn by the user. The seven cables that route from the mTPAD frame to the pelvis are configured such that two cables route to each of the two lateral extremes of the pelvic belt, and three cables route to the posterior extreme of the belt. The seven cables allow for the control of the six DOFs at the pelvis. Due to the configuration of the cable exit points on the rollator frame with respect to the pelvic belt, the maximum magnitude of solvable forces and moments per axis vary but are still appropriate for training forces of 10% body weight. Therefore, the force and moment profiles that the mTPAD can apply are customizable in both magnitudes and directions.

### B. Controller Design

**Fig. 2.**
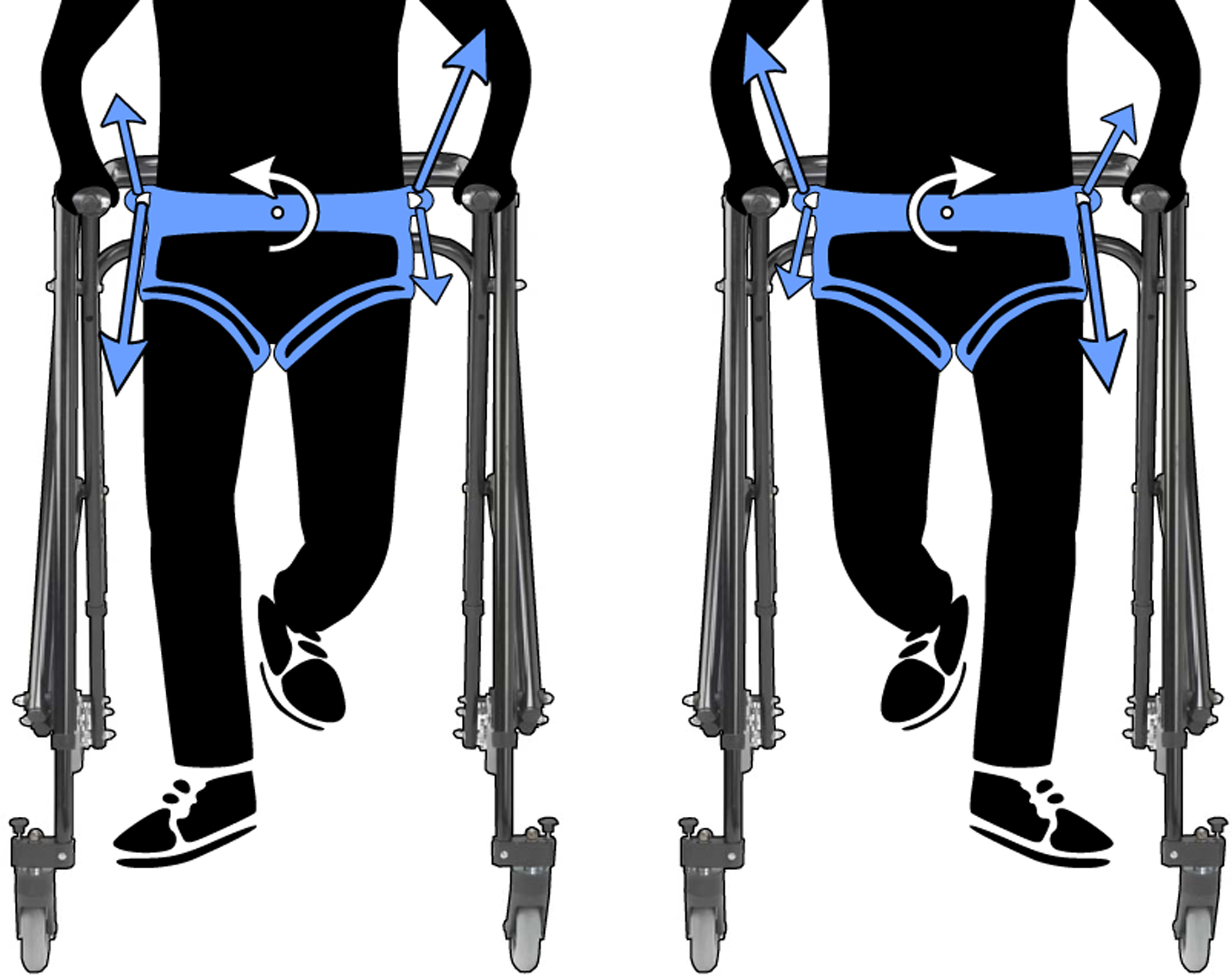
Cable tensions created frontal plane pelvic moments, depicted by white arrows around the pelvic center. Tensions, represented by the blue arrows with the relative size reflecting the relative cable tensions, are applied by cables routed from motor subassemblies on the walker to the pelvic belt worn by the user. The pelvic moments are synchronized with the phases of the user’s gait that correspond to single stance, i.e., a resultant upward force occurs on the contralateral pelvic extreme, and a resultant downward force is applied to the ipsilateral stance belt extreme. All seven cables are required to minimize all other forces and moments.

A frontal plane moment is applied to the pelvis for this work. This choice was motivated by frontal plane hip moments, which have a positive bimodal peak distribution from approximately 0% to 55% of the user’s gait cycle [33]. Considering both stance legs’ hip moments, these alternate in magnitude and direction. A simplification of this is a sine wave, with only one mode per stance leg. This sine wave simplification of a frontal plane pelvic moment is coordinated with the user’s gait such that the direction of the moment depends on which leg is in single stance, as shown in Fig. 2. The continuous gait cycle percentage was used to determine moment magnitude and direction to avoid any perturbative effects of instantly switching moment directions based on discrete gait phase stages like stance or swing. The continuous mapping requires real-time knowledge of where the user is in their gait cycle. The DeepSole system, an instrumented footwear developed in our lab [34], is used in this work to predict the gait cycle percentage of the user in real-time.

#### 1) Gait Phase Prediction:

The DeepSole system predicts the individual’s gait phase percentage in real-time using machine learning [31]. The DeepSole system’s gait phase prediction uses pressure and inertial measurement unit (IMU) data from both feet to predict the gait phase percentages of the left and right foot, where 0% corresponds to that foot’s heel strike, and 100% corresponds to the instance before the same foot’s next heel strike. The gait cycle percentage was based on the right foot for this application.

#### 2) Gait Phase - Moment Mapping:

**Fig. 3.**
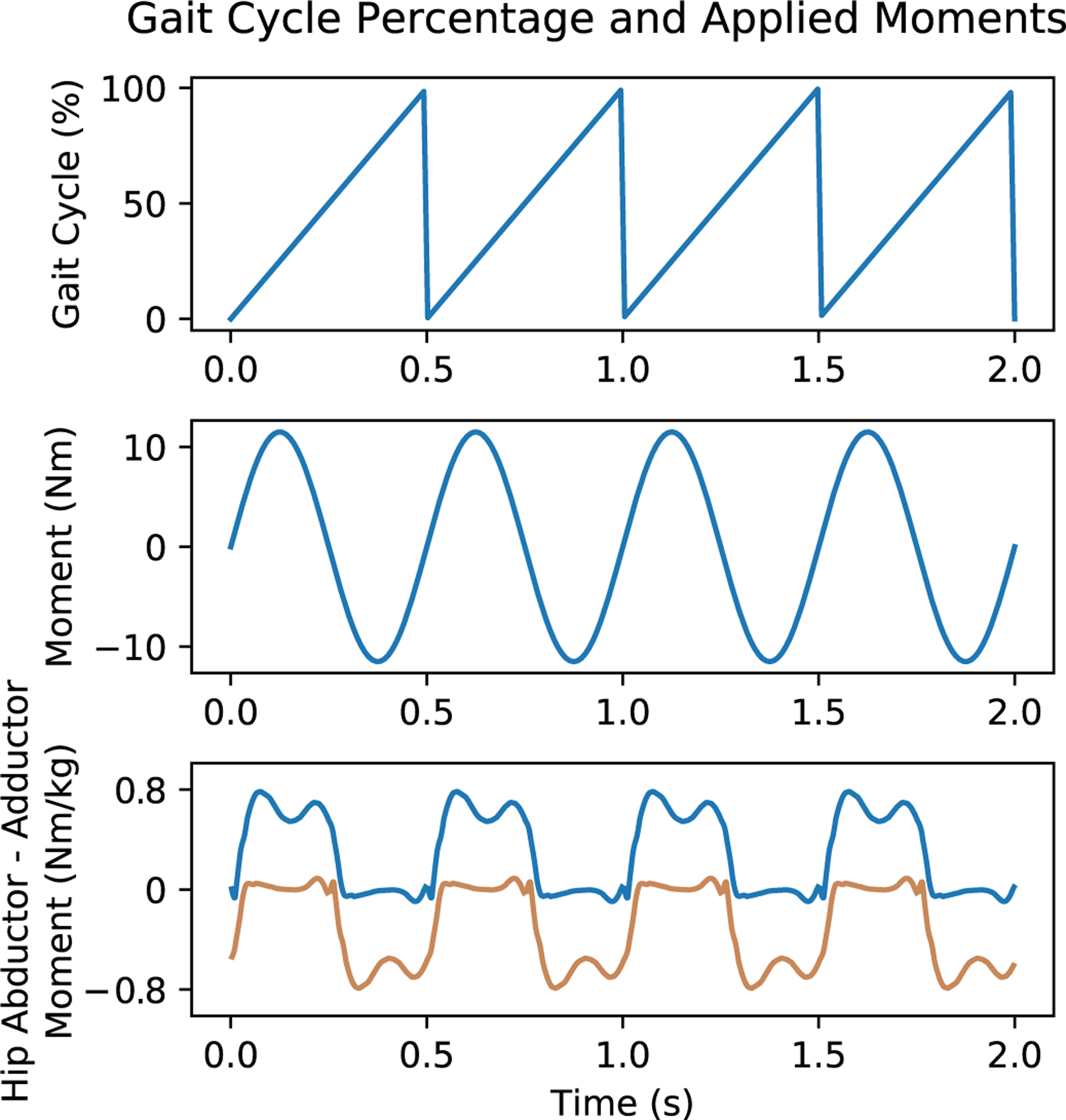
The applied pelvic moment is a function of the user’s right gait cycle percentage. Therefore one gait cycle, from the right heel strike to the next right heel strike, can include each moment direction during each leg’s stance phase. Zero moments are applied at 0% and 50% of the gait cycle percentage. Therefore, at each foot’s heel strike, the moment ramps up to its maximum and back down to zero before the ipsilateral heel strike, which would initiate the opposite moment direction. Shown in the bottom graph is the biological hip abductor-adductor moments as calculated in [33]. The blue illustrates the right hip with and the orange represents the left hip.

Consideration is required to coordinate the applied frontal moment with the gait cycle. The magnitude and direction of the frontal moment correspond to the stance leg during gait. However, care must be taken not to perturb the individual with a large change in moment magnitude from one time-step to the next. Therefore, a sinusoidal mapping of the gait phase percentage to the applied frontal moment was used to limit gait alterations caused by sudden changes in moment direction and magnitude, as shown in Fig. 3. Equation 1 was used to map the predicted gait cycle percentage to the applied pelvic moment:

(1) $$M_{y}=\left(.1*m_{BW}\right)*\left(0.5*w_{P}\right)*sin\left(\frac{2*\pi *p_{GC}}{100}\right)$$

where *M*_*y*_ is the frontal plane pelvic moment in N‧m, *m*_*BW*_ is the participant’s body weight in N, *ω_P_* is the participant’s pelvic width in m, and *p*_*GC*_ is the participant’s predicted right gait cycle output from the DeepSole system from 0 to 100%, as shown in the top of Fig. 3.

#### 3) Tension Optimization:

Once the goal moment has been calculated using Equation 1, the mTPAD’s high-level controller optimizes for the cable tensions using quadratic programming [32]. The following tension constraints are used in the quadratic programming scheme.

(2) $$\min\;f\left(T\right)$$

(3) $$f\left(T\right)=T^{⊤}T,$$

(4) $$JT_{ineq}=F_{ineq}$$

(5) $$T_{min}\leq T_{i}\leq T_{max};\;\;\;\left\{\begin{array}{l} -5N\;\;\leq \;\;F_{x,y,z}\leq 5\;N\\ M_{-goal}\;\;\leq \;M_{y}\leq M_{+goal}\\ -5N\cdot m\;\;\leq M_{x,z}\leq \;\;5\;N\cdot m \end{array}\right.$$

where *J* is the 6 × 7 system Jacobian, *T*_*ineq*_ is the optimized solution, *F*_*ineq*_ is the force-moment profile associated with the optimized tension solution, *T*_*min*_ = 1 N to ensure taut cables, and *T*_*max*_ = 35 N for safety.

## III Validation of the System

A dataset of overground walking under multiple conditions was collected from healthy individuals to demonstrate the continuous user-synchronized force application during overground walking. The human response to the frontal plane moments is also investigated. These conditions included walking without the mTPAD and walking with the mTPAD with and without pelvic moments. The experimental protocol and collected dataset were designed to shed light on the following: (i) How well the moment mapping corresponds to the actual gait phase percentage, (ii) If and how the position and orientation of the pelvis during gait are affected by the timed frontal moment, (iii) If and how the spatiotemporal gait parameters and muscle activity are affected by the timed frontal moment.

### A. Experimental Setup

**Fig. 4.**
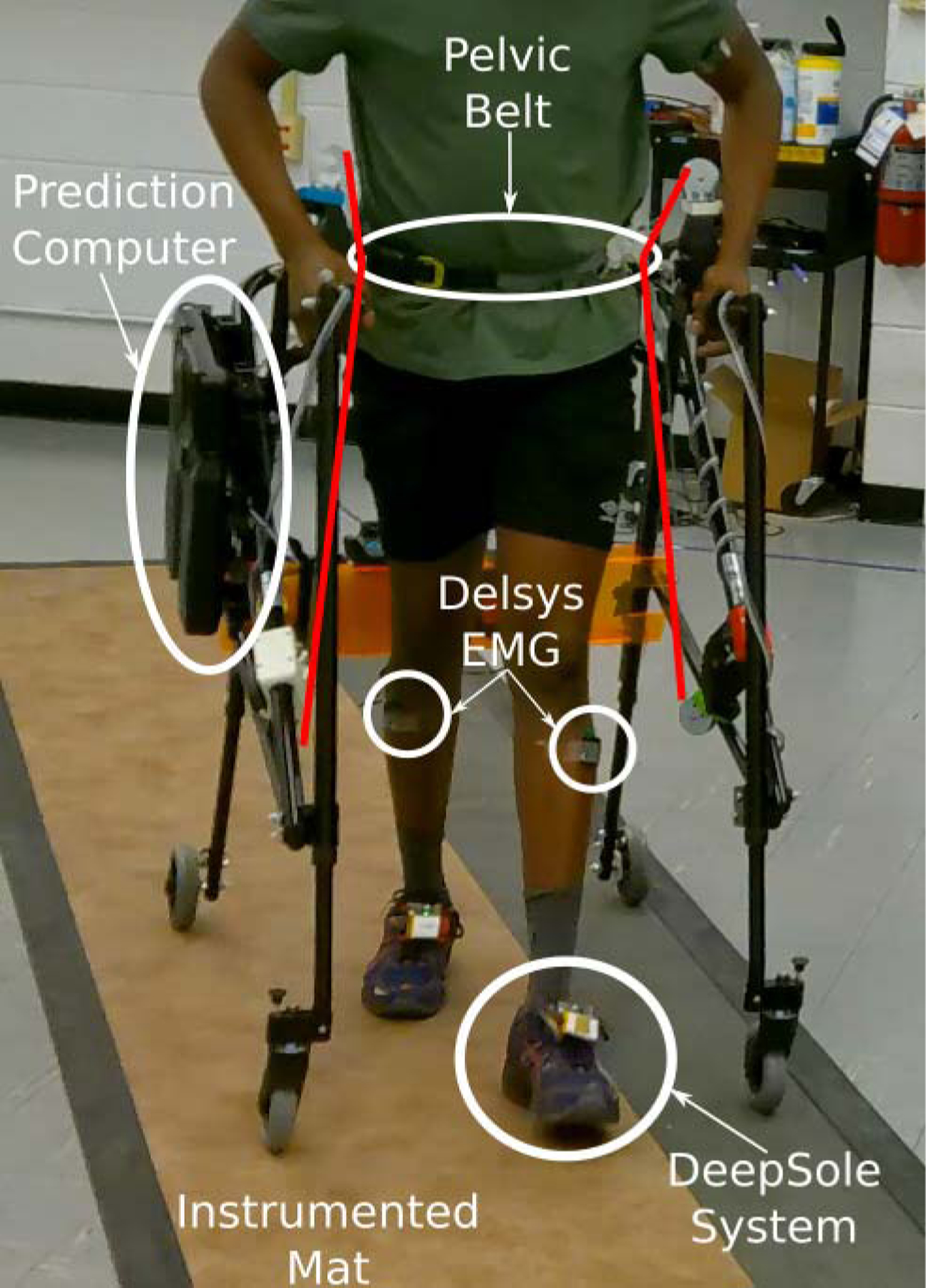
The experimental setup for mTPAD data collection. An MSI VR One is mounted to the frame of the mTPAD to run the DeepSole Gait Cycle Prediction. Delsys sEMG sensors record GM muscle activity (shown here on the Tibialis Anterior for illustration purposes only, as each user’s shorts cover sensors on the GM muscles). The instrumented mat records pressure beneath the feet of the individual as they walk overground. Cables (4 highlighted in red, 3 posterior cables occluded by user) that route from the mTPAD frame to the pelvic belt apply a force and moment to the pelvis as the participant walks.

Various data types were collected through a setup shown in Fig. 4 to answer these research questions. The left and right predicted gait cycle percentages were output from the DeepSole system at 40 Hz. The mTPAD recorded the local position and orientation of the pelvis, the goal force and moment profile at the pelvis, and the optimized tensions for each of the seven cables at 40 Hz. Delsys Trigno Avanti Sensors recorded surface electromyography (sEMG) signals from the left and right gluteus medius (GM) muscles at 2148 Hz. This muscle was selected for investigation as it assists in regulating pelvic obliquity during overground gait [5]. A Zeno Walkway recorded gait parameters at 120 Hz, which were used to calculate spatial and temporal gait parameters. All of these datasets were time-synchronized using a custom sync box. The sync box receives a transistor-transistor logic (TTL) signal from the Zeno Walkway, which is routed to the sEMG system, and a User Datagram Protocol (UDP) packet is sent to the mTPAD and DeepSole through WIFI.

### B. Protocol

This experiment was completed by 10 healthy participants (age: 28±3years, height: 169±11.3cm, weight: 72.3 ± 8.4kg). Before participation, all participants were informed of the following procedure and given the opportunity to ask questions. Each participant subsequently signed a written consent form approved by the Institutional Review Board of Columbia University. Before beginning, each participant was fitted with a pair of athletic shoes housing the DeepSole system. Whole numbers of shoe sizes were available, and snug yet comfortable lacing was ensured. The pelvic belt was placed at the level of the iliac crests, and the walker height was self-selected and adjusted between 36–41 inches.

The experiment protocol included the following conditions: (i) Baseline: overground walking without mTPAD, (ii) mTPAD Baseline: overground walking with the mTPAD, with no external forces applied to the pelvis, and (iii) mTPAD Moments: overground walking with the mTPAD, with a frontal moment applied. Conditions (i) and (ii) were five minutes each, and condition (iii) was fifteen minutes. After condition (ii), a short break was given to each participant so that the gait phase prediction model could be retrained with their baseline data.

For all three of these conditions, participants walked in a stadium pattern with the Zeno Walkway aligned with one of the stadium-shape straightaways. Therefore, participants would walk straight down the mat, turn towards their right side after exiting it, then walk back parallel to the mat. This pattern ensured that participants walked continuously and did not stop throughout each condition duration. For all participants, the following (avg ± std) of laps and steps were included in training and analysis per condition: Baseline, 21.1 ± 2.8 laps with 196.0±19.7 steps; mTPAD Baseline, 15.3±2.1 laps with 162.0 ± 17.3 steps; mTPAD Moments, 41.7 ± 8.2 laps with 459.2 ± 99.5 steps.

After both Baseline conditions were completed, the gait mat data were processed using the PKMAS software. These data and the time-synchronized raw DeepSole IMU and pressure data were added to the training dataset, and the prediction model was retrained for 50 epochs. Thus, the training data set included data from the participant as well as all prior participants, i.e., participant N’s model was trained with the baseline data from participants 1, 2, … , and N. This strategy was adopted because the prediction model presented in [31] did not include data from individuals walking inside the mTPAD. We aim to tailor the prediction to each individual’s gait by retraining the model with their baseline data.

### C. Data Analysis

Once data were collected, the gait cycle prediction and timed moment applications were characterized. The changes in pelvic kinematics and GM response were evaluated.

#### 1) Segmentation:

All cyclic data were segmented and averaged to get a representative gait cycle per data type per condition per subject. The foot pressures, spatial, and temporal gait parameters were calculated and output using the ProtoKinetic software, PKMAS [35]. Left and right heel strikes were defined as the instants left and right maximum foot pressures became non-zero.

#### 2) Moment Application Characterization:

The gait cycle percentage from the mat was time normalized using the right heel strikes; 0% corresponded to each heel strike, and 100% corresponded to the instant before the next right heel strike, assuming a constant gait cycle percentage increase. The right predicted gait cycle percentage from the DeepSole and the calculated frontal moment output by the tension optimization were segmented using the timestamps of the gait mat’s right heel strikes and interpolated to 100 points.

#### 3) Pelvis Kinematics Data:

**Fig. 5.**
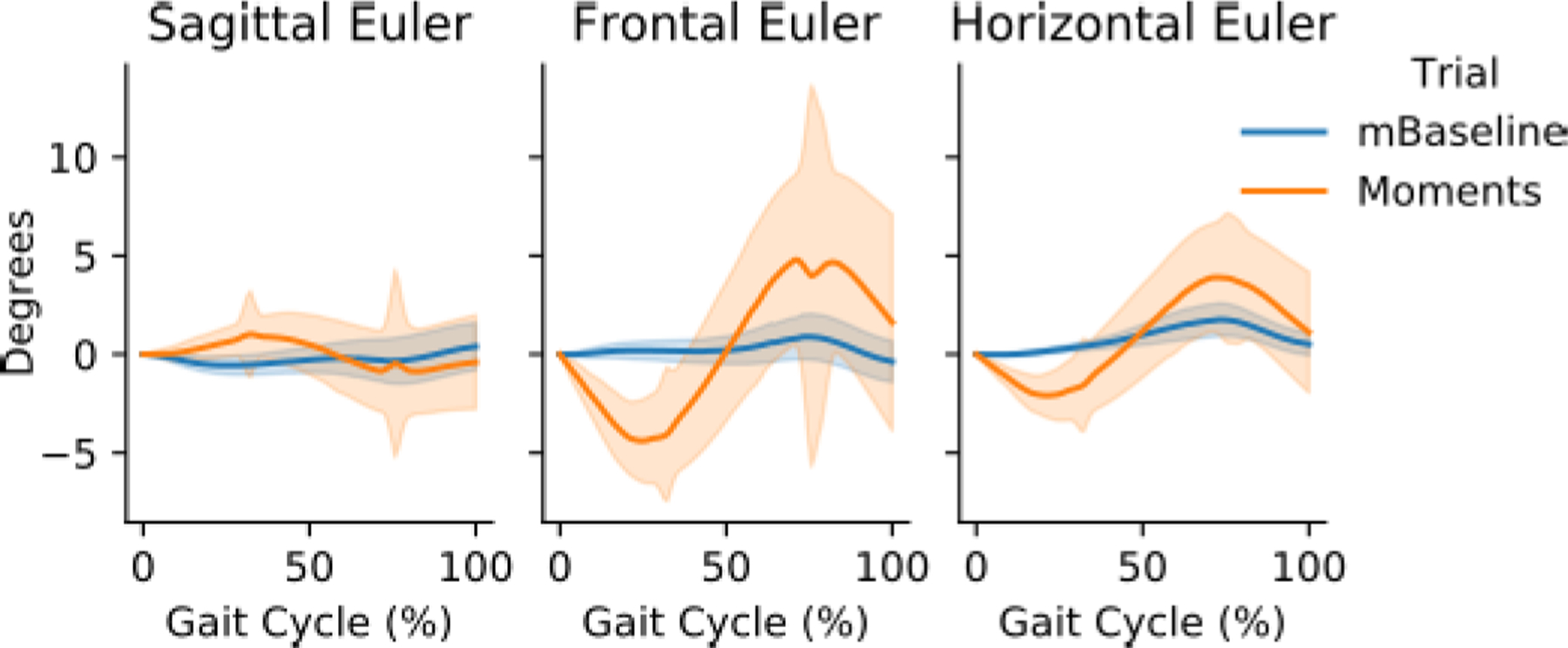
Example pelvic angles for one study participant. The orange represents the trial where frontal moments are applied, and the blue represents the trial where no frontal moments are applied. The solid line represents the mean of all gait cycles, and the shaded region represents the 95% confidence interval. Each graph is in degrees, and from left to right the Euler angles are taken in the pelvic sagittal, frontal, and horizontal planes. Peaks in the confidence interval represent cable configurations with more than one viable solution to the forward kinematics problem. Geometrically, there are eight solutions, but many of these lie outside the walker frame, so we can eliminate them. However, there are segments where two solutions may exist in the workspace, causing a temporary jump in the pelvic tracking.

The mTPAD forward kinematics pelvic trajectories were segmented using right heel strikes. Segments were interpolated to 50 points and averaged for each condition per subject. An example of one person’s representative data is shown in Fig. 5. The ranges of pelvic rotations were calculated per segmented gait cycle and averaged per condition and participant to compare the changes in pelvic motion across conditions.

#### 4) sEMG Data:

**Fig. 6.**
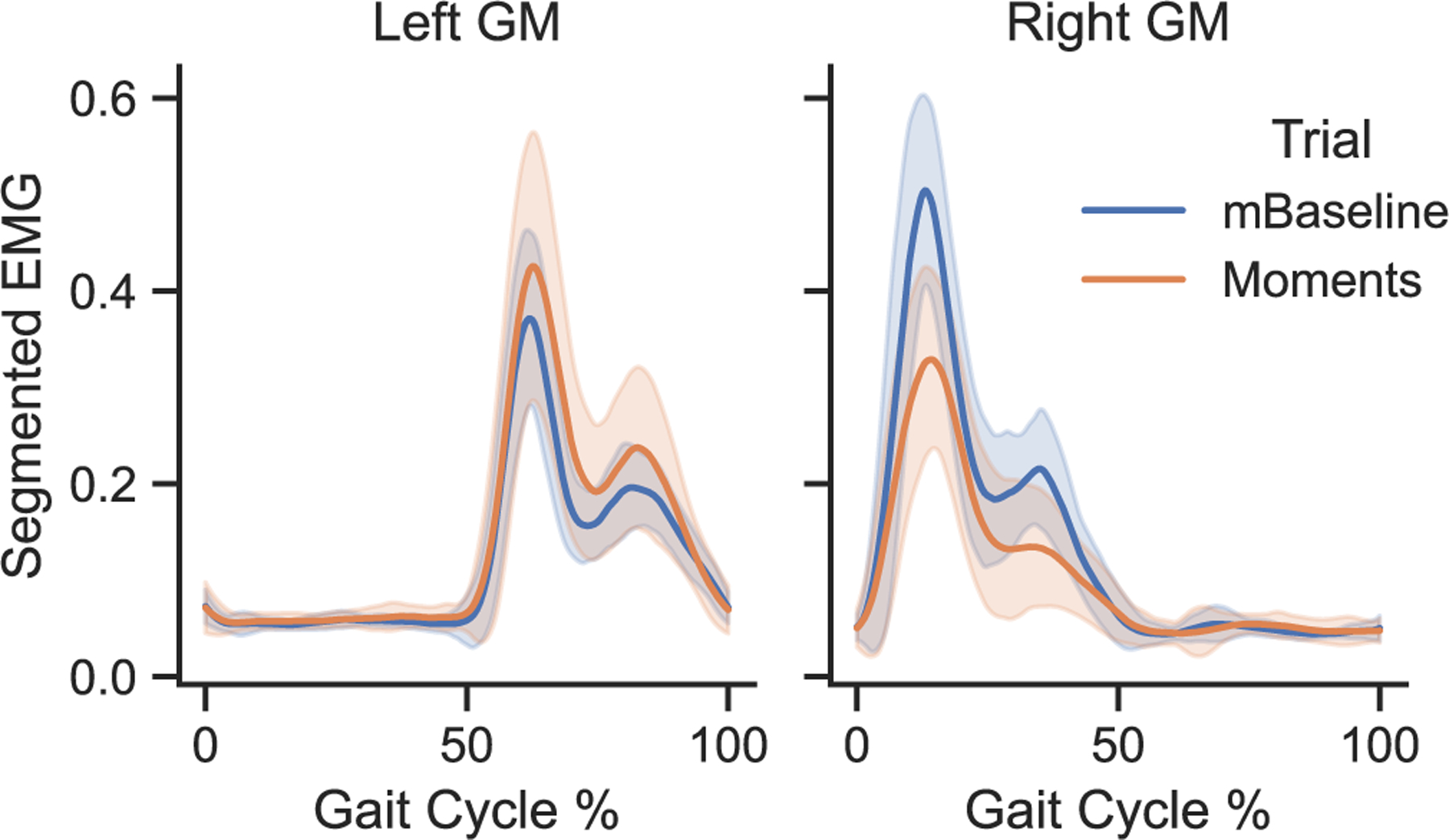
Example GM sEMG data from one subject. Both subgraphs are segmented with the right heel strike representing 0% of the gait cycle. The blue with shading represents the mTPAD trial with no frontal moments, and the orange with shading represents the mTPAD trial with frontal moments. Each solid line represents the average signal for all laps of this participant, and the shaded regions around the solid lines represent the standard deviation associated with the mean signal.

Raw sEMG signals were detrended, bandpass filtered, enveloped, and low pass filtered using Delsys EMGworks Analysis. Both the bandpass (20 Hz and 450 Hz) and low pass (5 Hz) filters [36], [37] were second-order Butterworth filters applied in both direct and reverse signal directions to avoid phase distortions, therefore being 0-phase, fourth-order filters. Processed sEMG signals were normalized across all trials with the maximum value for each sensor. The normalized signals were segmented per stride, using right heel strikes as 0% and the moment before the next ipsilateral heel strike as 100%. Stride segments were interpolated to 500 points and averaged per condition per subject. An example of these representative cycles is shown in Fig. 6. Each segmented cycle was integrated, and integrated sEMG (iEMG) values were averaged per condition and participant to compare the changes in GM activation per condition.

### D) Statistical Analysis

The mTPAD Baseline data are compared to the mTPAD Moment data to evaluate the effects of the timed frontal moment on pelvic kinematics and GM muscle response. Before selecting the appropriate statistical test, the normality of the distributions of each data was determined using a one-sample Kolmogorov-Smirnov normality test. The Wilcoxon signed-rank test was used when significantly different from a normal distribution. One-way repeated measure analysis of variance (RM-ANOVA) tests were used when not significantly different from a normal distribution. All tests were run using Python Statsmodels [38] and Scipy Stats, and statistical significance was defined as *p <* 0.05. For statistical comparisons, the following notation is used: *: *p <* 0.05; **: *p <* 0.01; and ***: *p <* 0.001.

## IV Results

### A. Moment Characterization

**Fig. 7.**
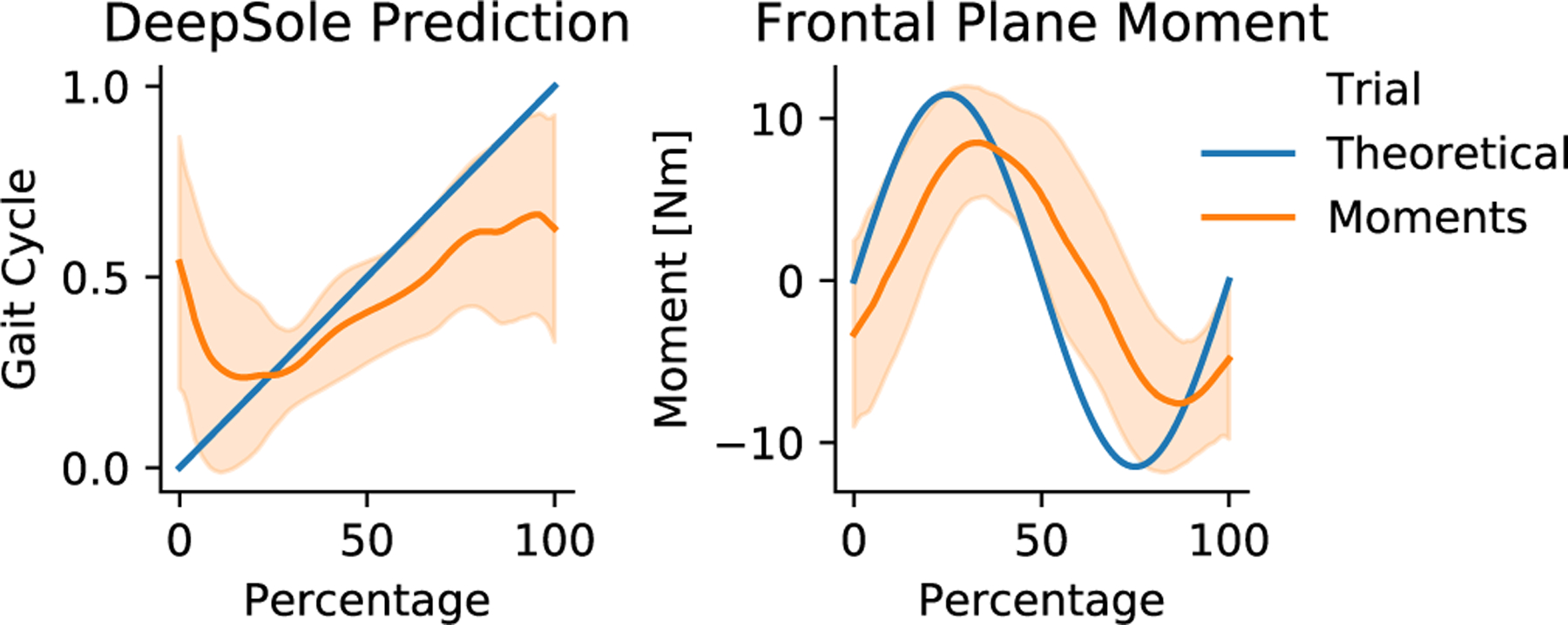
The DeepSole gait cycle prediction and applied frontal plane moment. The x-axes of both graphs represent the gait cycle percentage segmented by right heel strikes. Solid lines represent all participants’ group means of all cycles, and the shaded region represents the 95% confidence intervals. Left: The DeepSole right gait cycle prediction is shown in orange, and the right gait cycle percentage from the gait mat is shown in blue. Right: The optimized frontal moment as calculated by the mTPAD’s tension solver is shown in orange. The sine transform of the mat segmented gait cycle percentage is shown in blue. The amplitude of this sine wave is the average of all participant’s amplitudes.

The predicted gait cycle percentage from the DeepSole system and the mTPAD’s output frontal plane moment are shown in Fig. 7. The phase shift between the theoretical frontal moment based on Equation 1 and the mTPAD’s calculated output is 10%. The (mean ± sd) for each of the parasitic forces and moments, i.e. the secondary forces that were minimized during optimization, for all participants’ timed moments sessions are *F*_*x*_ : (−0.06 ± 1.3) N; *F*_*y*_: (1.4 ± 1.6) N; *F*_*z*_: (0.6 ± 0.3) N; *M*_*x*_ : (−0.3 ± 0.1) N·m; and *M*_*z*_ : (−0.1 ± 0.2) N·m.

### B. Pelvis Kinematics

**Fig. 8.**
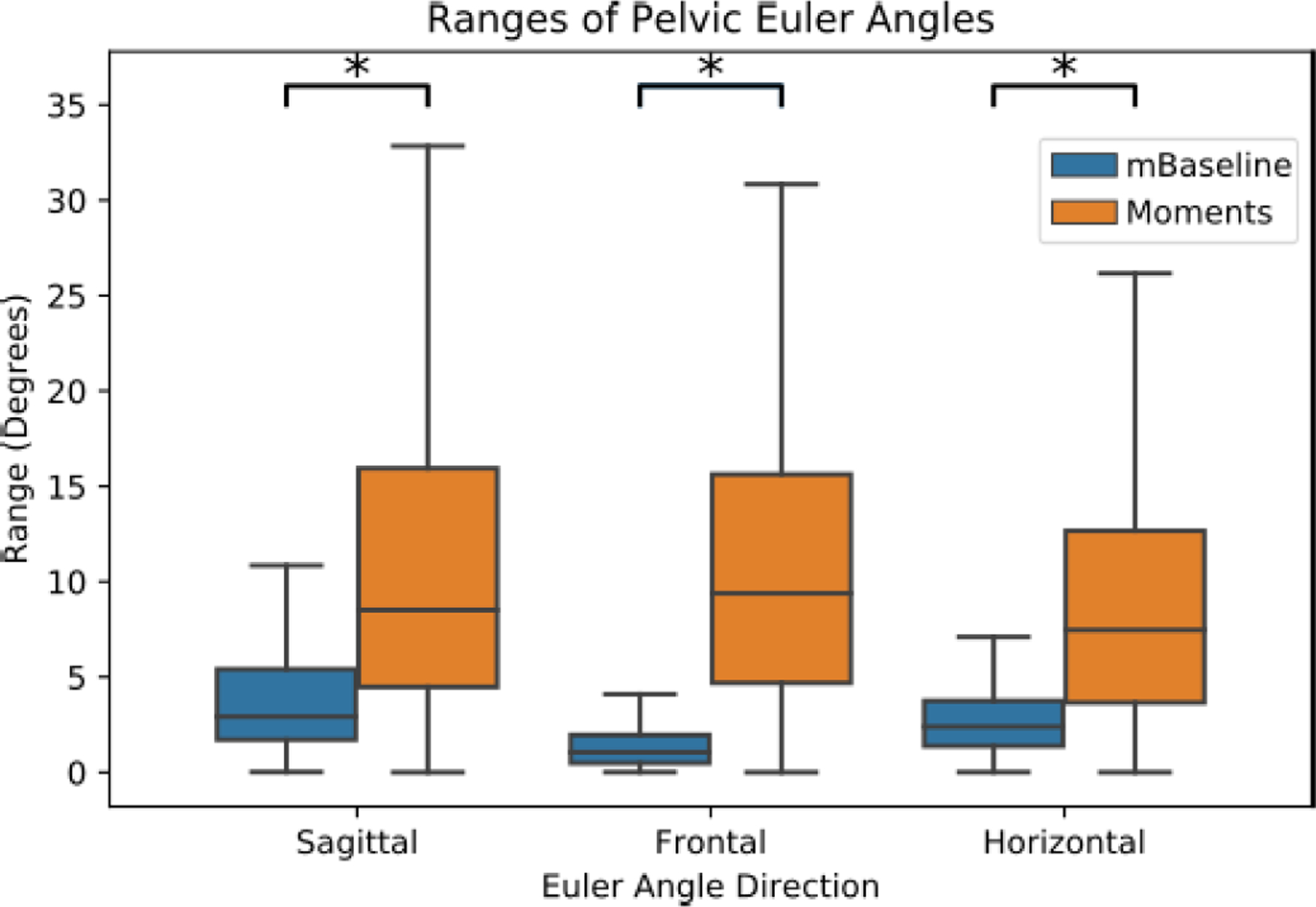
Range of values per stride for all strides and all participants. The x-axis represents the three Euler angle directions, and the y-axis represents the range of degrees per stride. The mTPAD trial where no moment is applied is shown in blue, and the orange boxes represent the mTPAD trial with frontal moments applied.

The differences in Euler angles with and without the applied moment were evaluated to determine if the frontal plane moments significantly affected the pelvic range of motion. The distributions for the pelvic rotational ranges per cycle are shown in Fig. 8. For all directions, participant mean distributions for the mTPAD Baseline condition were significantly different than a normal distribution, so Wilcoxon signed-ranked tests were used. For Euler angle directions, the ranges of values are significantly higher when a frontal plane moment is applied. For the sagittal plane, the corresponding group average Euler angle is significantly higher while the mTPAD is applying a frontal plane moment (M = 10.1°, SD = 10.6°) than when no moments are applied (M = 4.6°, SD = 5.8°); Z = 2, p = 0.025. The Euler angle corresponding to the frontal plane is significantly higher while the mTPAD is applying a frontal plane moment (M = 9.9°, SD = 10.0°) than when no moment is applied (M = 2.6°, SD = 5.6°); Z = 0, p = 0.0117. The Euler angle corresponding to the horizontal plane is significantly higher while the mTPAD is applying a frontal plane moment (M = 8.3°, SD = 9.2°) than when no moment is applied (M = 3.0°, SD = 2.4°); Z = 1, p = 0.0173.

### C. sEMGs

**Fig. 9.**
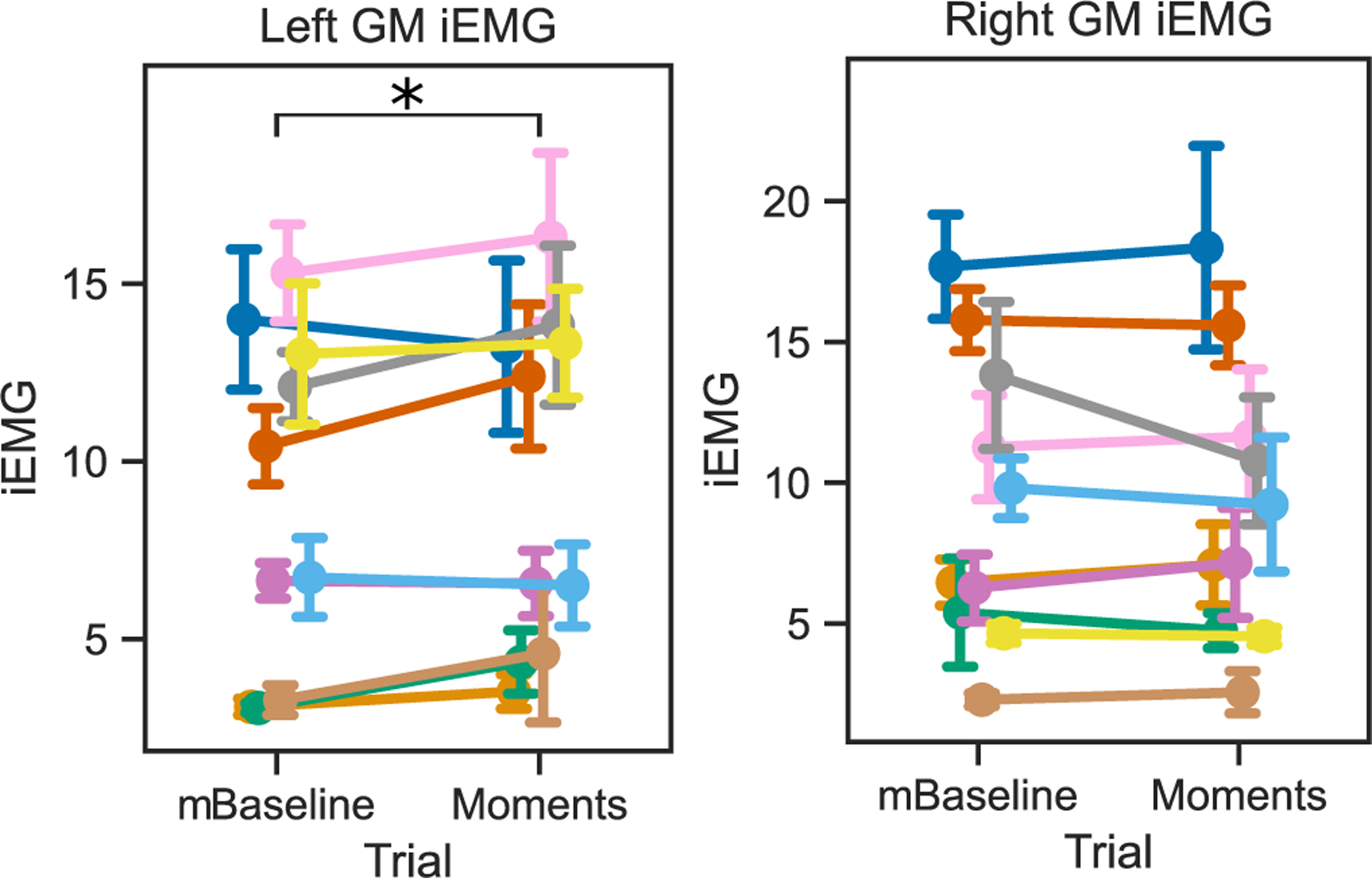
Group results of iEMG values per step for the left and right GM. mBaseline shows the mean and standard deviation iEMG values per subject in the mTPAD Baseline condition. Moments shows the mean and standard deviation iEMG values per subject in the mTPAD Moments condition. Each participant’s mean value per condition was used in the one-way RM-ANOVA described in III.D.

The differences between the mTPAD Baseline and Moment conditions were evaluated to determine if the frontal plane moments significantly affected the iEMG values for the left and right GM muscles. The GM muscles are considered pelvic stabilizers. They are primarily active during the ipsilateral leg’s stance, i.e., the contralateral leg’s swing [39], and have higher activations when individuals experience pelvic perturbations in the frontal plane [40]. The distributions of the left and right GM iEMG values per participant are shown in Fig. 9. The left and right means per participant for these iEMG values were normal for both conditions, so one-way RM-ANOVAs were used. For the right GM, there was not a significant difference between the mTPAD Baseline (M = 9.19, SD = 5.18) and the mTPAD Moments (M = 8.81, SD = 5.25) conditions; F(1,9) = 0.23, p = 0.64. For the left GM, there was a significant difference between the mTPAD Baseline (M = 8.55, SD = 4.71) and the mTPAD Moments (M = 9.10, SD = 4.83) conditions; F(1,9) = 5.94, p = 0.038.

## V Discussion

Implementing a continuous moment-based controller using a novel overground gait training device, the mTPAD, is essential for user-in-the-loop gait training with overground control of pelvic movement. The effects on the mTPAD user’s gait highlight the capabilities of the mTPAD’s range of applications. Most overground devices only focus on bodyweight augmentation, i.e., applying an upward force to unload the individual. However, the possibility for mTPAD’s force application at the pelvis is not limited to the vertical axis. This device’s range of force and moment application is illustrated by demonstrating that frontal plane moments can be applied. The type of force applied by the mTPAD has the flexibility of direction, and the mTPAD can alter the applied pelvic forces and moments based on the user’s gait in real-time. This novel control of an overground gait training device can target specific kinematics and elicit higher muscle activation, tailoring gait training to the user.

When a frontal plane moment is applied by the mTPAD device based on the predicted gait cycle percentage as output by the DeepSole system, the phase shift error is 10% of the gait cycle. Previously, the DeepSole system has been shown to have a root mean square error of 7.2% of the gait cycle percentage [31]. Therefore, utilizing the predicted gait cycle percentage in the mTPAD’s controller to calculate the cable tensions minimally increases the delay. Synchronizing the applied pelvic forces to the gait cycle percentage using the DeepSole system is a new capability that explores various forcing functions of the gait cycle percentage. Various forces and moments can now be applied continuously at specific gait phases, and these effects leave much to be explored.

The possibilities for different functions and their goals are vast. Various wave transformations like a square wave, triangle wave, etc., could be implemented, or a combination of multiple sinusoidal functions could create asymmetrical or pseudorandom wrenches. These forcing functions can be motivated by specific gait needs, from applying larger forces towards one side to train individuals with an asymmetric gait to applying forces during smaller time windows to introduce variability or affect specific gait phases.

During the frontal plane intervention, all three pelvic Euler angles experienced significant increases when a frontal plane moment was applied. These increases in the range of motion for all three pelvic angles are at least 5°. These ranges are considerable, since the pelvic ranges for overground gait are around 3°, 10°, and 10° for pelvic tilt, obliquity, and rotation, respectively [41]. These increases could be significant for children with CP, who tend to lose pelvic and hip static range of motion as they age [42], especially since a restriction of pelvic movement can decrease the range of motion of the knees and ankles [43]. Initially, it was hypothesized that only the angle corresponding to the pelvic obliquity would be affected, as this is the primary plane of the wrench application. However, applying a pure frontal plane moment altered all pelvic rotations. The pelvic rotations may be coupled because the pelvis is a segment that does not rotate around its center but about the stance leg’s ipsilateral hip socket. Research has shown that this coupling is more prominent when the rotation is along the frontal and horizontal planes [44]. Other kinematic alterations which may have resulted from the applied moments could also contribute to the coupled pelvic rotations. Alterations in the lower limb or torso kinematics could also result in these coupled motions. While the mTPAD doesn’t constrict the movement of the lower limbs and is a fully portable system, information on the lower limb angles and trunk range of motion are not recorded. Motion deviations of these proximal segments to the pelvic segment could also influence the other pelvic angles while the frontal plane moments are applied. It is also possible that a different pelvic force and moment profile would better isolate one pelvic motion. Perhaps by applying a moment about multiple planes, it would be possible to isolate a change in only one pelvic direction, but this would need to be explored. The parasitic forces and moments in the nonfrontal plane could also affect the other directions. However, these forces and moments are bound to 5 N or 5 N‧m for each force and moment.

The application of a frontal plane moment was selected to highlight the capabilities of the mTPAD device. Since the GMs are considered pelvic regulators, we investigate the activation of the GMs while the frontal plane moments are applied. A significantly higher activation was found for the left GM when a frontal plane moment was applied. This increase, rather than a decrease that may be expected when provided an assistive moment, could be due to the participants acting against this added moment to try and maintain their natural gait. However, this increased activation was not seen bilaterally with the right GM. This unilateral adaptation to the applied forces could be due to error in the applied moments. The inaccuracy of the DeepSole prediction of the gait cycle percentage is most prominent at the bounds of the segmented gait cycle, i.e., where the right heel strikes occur. The applied frontal plane moment would increase in magnitude after the right heel strike to affect the right GM, so any errors in the prediction around this event could affect the moment application. Perhaps also, more granular changes to the cyclic GM response could have been overlooked by considering the GM activation over the entire cycle. A deeper understanding of the GM response could be attained by analyzing subsections of the gait, such as Mid versus Terminal Stance. Other sEMG analysis methods could also be used, such as Statistical Parametric Mapping (SPM), which considers inter-muscle and time dependency of multiple sEMG signals [45].

The differing responses between the left and right GM muscles could also result from biomechanic compensations. It is possible that the footedness or handedness of the study participants, being mostly right dominant, played a role in this asymmetric moment compensation. If participants favor their right side, they may brace themselves with their right arm or have a stronger right GM muscle. Also, when a constant downward force is applied at the pelvis, users load the mTPAD handrails, which alters the force distribution through the feet [32]. Investigating the loading strategy by recording the muscles in the arms or the forces between the hands and the walker grips could shed light on compensatory strategies. Using a sine wave to transform the gait cycle percentage also includes a ramp up and down of the applied moment, meaning the maximum moment happens for a short period of the gait cycle. This short maximum moment duration could also affect the amount of activation change in the pelvic regulators.

To fully understand the compensatory strategies taken by the participants, it would be necessary to know how the individual loads the frame while walking. However, the current mTPAD setup does not include sensors to measure the interaction between the hands and the frame. This limitation restricts the understanding of how the applied pelvic forces are distributed through the arm handles, which requires further investigation. If pressure sensors are added to the handles, a biofeedback aspect could be incorporated to encourage users to place less weight on the frame. The device’s width is wider than the instrumented walkway, so the pressure beneath two of the four wheels cannot be measured. We could gather more insight into the participants’ loading strategies during different force and moment applications by adding sensors to measure these pressures. It would also be interesting to apply other wrenches to determine which best target specific muscles for strengthening and training. This could motivate training paradigms for different patient populations with specific muscle weakness, like those with cerebral palsy who experience Trendelenburg gait due to pelvic regulation defects. Special care may be needed for the gait cycle prediction algorithm for patient populations with higher gait variability. While in this case, it worked for a group of neuro-typical individuals, the accuracy may decrease when implemented with those with irregular gait. However, there is much potential for predicting a variety of users’ gaits. By tailoring various interventions to the gait cycle of the individual users, this device has great potential for training people based on their specific needs and deficiencies.

Applying a moment about the pelvis while an individual walks overground allows future investigation of a range of interventions. Not only is the direction/plane of the force/moment applied at the pelvis flexible, but different transformations from gait cycle percentage to the desired wrench can also be explored. The ability to modify the targeted pelvic kinematics and muscle activations motivates user-specific interventions, which can improve the potential for overground gait training.

## VI. CONCLUSION

This work demonstrated the efficacy and effects of an innovative overground mTPAD and its novel human-in-the-loop controller that synchronized gait cycle percentage to frontal plane pelvic moments. We showed that while applying frontal plane pelvic moments overground, the range of pelvic angles in the sagittal, frontal, and horizontal planes increased. The applied pelvic moment and altered pelvic kinematics also increased the left GM muscle activation, associated with controlling pelvic obliquity. This work opens the door to tailoring a gait training intervention to individuals’ specific needs while also considering their gait in real-time. This allows for various pelvic forces and moments to be studied during different segments of an individual’s gait, putting the individual at the center of the intervention design.

Individuals with limited control or coordination of pelvic obliquity during ambulation, such as those with CP who exhibit Trendelenburg gait, require physical therapy or other interventions to limit associated degeneration of muscle strength or gait patterns [46]. Trendelenburg gait is an abnormal gait pattern classified by a dropping of the pelvis to the contralateral side while walking and is caused by a weakened gluteal musculature [47]. Strengthening muscles that assist with pelvic stabilization during single stance could improve coordination for these individuals [48]. These individuals may benefit from this novel training paradigm. It may strengthen the muscles associated with pelvic obliquity control while also assisting the individual by limiting pelvic drop during swing, allowing for repetitive practice of a more regular gait pattern.
